# Management and Follow-Up of Biventricular Thrombi

**DOI:** 10.7759/cureus.39269

**Published:** 2023-05-20

**Authors:** Wahab J Khan, Muhammad Asif, Ifrah Nadeem, Megan Moeding, Thomas Baab, Mohammed Chowdhury

**Affiliations:** 1 Internal Medicine, University of South Dakota Sanford School of Medicine, Sioux Falls, USA; 2 Internal Medicine, Avera McKennan Hospital and University Health Center, Sioux Falls, USA; 3 Cardiovascular Medicine, North Central Heart Institute, Sioux Falls, USA

**Keywords:** rivaroxaban, treatment review, direct oral anticoagulants (doac), biventricular thrombus, viral cardiomyopathy

## Abstract

A thrombus is the most common intracardiac lesion. Isolated thrombi usually occur in the setting of ventricular dysfunction, such as a dyskinetic or hypokinetic myocardial wall, following an acute myocardial infarction (MI) or in cardiomyopathies (CM). Concurrent biventricular thrombus formation is rare. There are no clear guidelines for the treatment of biventricular thrombus. In this report, we describe our experience of the successful treatment of a case of biventricular thrombus with warfarin and rivaroxaban.

## Introduction

Intracardiac lesions are not uncommon. These could be broadly categorized into neoplastic and non-neoplastic masses. The non-neoplastic category includes mainly vegetation and thrombi [1]. An intracardiac thrombus is the most common lesion of all these. Isolated thrombi more commonly occur in the right or left ventricle (LV) and are associated with ventricular dysfunction. Concurrent biventricular thrombi formation is rare. It usually happens in the presence of prothrombotic states such as protein C and S deficiency, heparin-induced thrombocytopenia, antiphospholipid syndrome, and hypereosinophilic syndrome [2]. In this report, we discuss the management of concurrent biventricular thrombi that were likely triggered by viral myocarditis in the setting of non-ischemic cardiomyopathy (CM) and severe LV dysfunction.

## Case presentation

A 35-year-old African American male presented with increasing shortness of breath and non-radiating midsternal pressure-type pain for two days. A review of systems was significant for abdominal bloating, decreased appetite, nausea, vomiting, and diarrhea for a similar duration. His medical history included anxiety, depression, and longstanding polysubstance abuse of opiates, cocaine, and marijuana, with previous inferior myocardial infarction (MI) secondary to substance abuse. He had an underlying baseline ejection fraction (EF) of 45-50%, mild global hypokinesis of the LV, and a previous angiogram revealing a right dominant system with normal coronary arteries. His medications included aspirin, clopidogrel, and diltiazem. Initial vital signs included a BP of 140/105 mmHg, HR of 120 beats/minute, SaO_2_ of 94% on room air, a temperature of 97.9 °F, and a BMI of 19 kg/m^2^. Physical examination showed a young cachectic male in mild respiratory distress, mildly elevated jugular venous pressure (JVP), fine bibasilar end-inspiratory crackles, and a trace pedal edema without loud murmur or abdominal tenderness.

**Figure 1:**
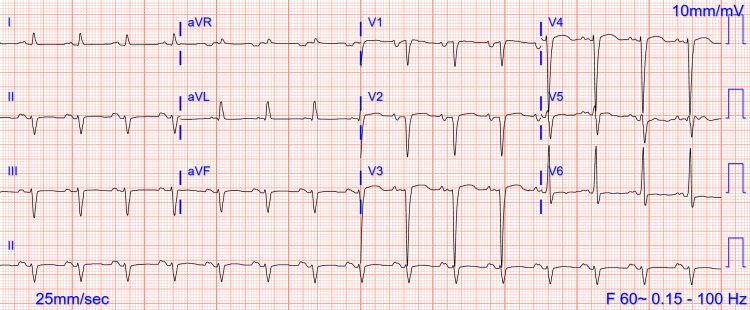
EKG showing sinus rhythm, left atrial enlargement, left anterior fascicular block, and an old anteroseptal infarct EKG: electrocardiogram

**Figure 2:**
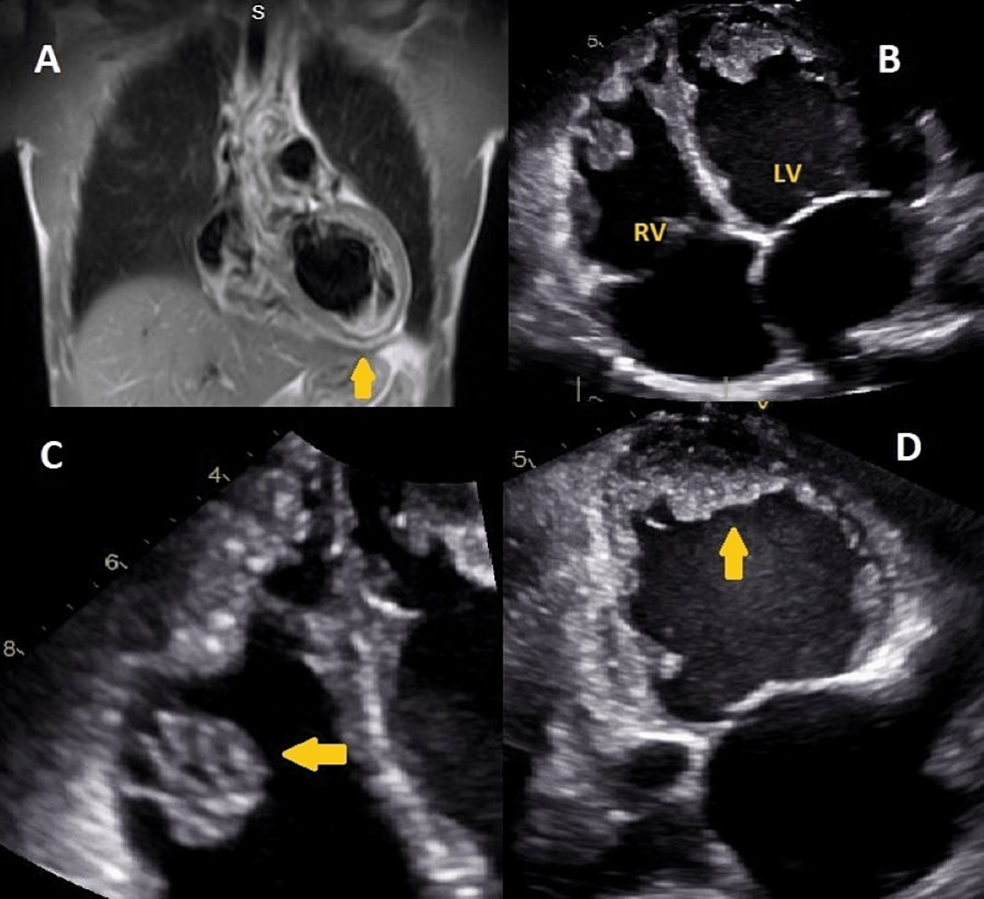
Cardiac MRI with myocarditis findings and echocardiogram done a few months later showing biventricular thrombus (a) Cardiac MRI showing nonspecific mid-myocardial enhancement consistent with myocarditis. (b) Transthoracic echo showing left and right ventricular thrombus along with severe dilation. (c) Right ventricular apical thrombus. (d) Left ventricular mural thrombus MRI: magnetic resonance imaging

On admission, his basic labs showed normal hemoglobin and electrolytes. Other remarkable labs included a C-reactive protein (CRP) level of 4 mg/dL (normal: <0.5 mg/dL), troponin value peaked at 1.24 ng/mL (normal: <0.03 ng/mL), and a B-type natriuretic peptide (BNP) level of 1752 pg/mL (normal: <100 pg/mL). An EKG showed a left anterior fascicular block, and a previous anteroseptal infarct (Figure 1). Transthoracic echo (TTE) showed EF of 25-30% and mildly dilated LV with severe global hypokinesis. Cardiac MRI (CMR) showed severe global hypokinesis and nonspecific mid-myocardial enhancement consistent with myocarditis or non-ischemic cardiomyopathic pattern (Figure 2a). Among the viral serologies and respiratory viral panel, only Coxsackie type b4 virus serology was positive. In addition to aggressive diuresis for acute heart failure, he was treated for pericarditis and myocarditis with colchicine and ibuprofen, with improvement in symptoms. He was discharged on carvedilol, lisinopril, furosemide, spironolactone, and aspirin, along with a life vest. The patient presented again with progressively worsening dyspnea after two months and was admitted for acute on chronic heart failure and cardiogenic shock. A repeat TTE at that time showed EF ~10%, 4.2 x 2.1 cm LV apical thrombus (Figures 2b, 2c), and global hypokinesis of LV with severe dilation (Figure 2b). The right ventricle (RV) was moderately dilated with a 2.2 x 1.8 cm mid-RV and a second RV apical thrombus (Figures 2b, 2d). He was started on a heparin drip and acute congestive heart failure (CHF) exacerbation treatment and discharged on warfarin after six days of hospital stay.

## Discussion

Intracardiac thrombi are associated with the same principle of thrombogenesis as anywhere else in the body, i.e., Virchow's triad; (a) stasis [dys- or hypokinesis as in CM, myocardial stunning as in MI], (b) hypercoagulability (e.g., post-MI inflammation, factor V Leiden and acute myeloid leukemia), and (c) endocardial injury (post-MI, eosinophilic myocarditis, etc.) [3].

The LV thrombus formation is more common after ST-elevation myocardial infarction (STEMI) than non-ST-elevation myocardial infarction (NSTEMI), highlighting the interplay of Virchow's triad in STEMI, which includes stasis (hypokinesis, akinesis, or dyskinesis), endocardial injury, and lastly, post-MI inflammation in the ischemic milieu [4]. An increased mean platelet volume, CRP, and fibrinogen levels after MI leads to LV thrombus formation. The second most common cause of LV thrombus is non-ischemic or dilated cardiomyopathy (DCM). It includes non-compaction, hypertrophic, peripartum, arrhythmogenic, and Takotsubo cardiomyopathies. Low LVEF with global hypokinesis associated with scarring is the risk factor for thrombosis in these patients [4]. Other less common causes include amyloidosis, myocarditis, and hypercoagulable states such as malignancies.

LV thrombi can be divided into the mural and protuberant thrombi. A mural thrombus, or laminated thrombus, is usually adjacent to the endocardial surface. A protuberant, also called a mobile thrombus, is generally distinct from the adjoining endocardium and protrudes into the cavity. Contrast-enhanced echo has more sensitivity for detecting mural thrombi than non-enhanced imaging. However, CMR offers the best sensitivity and specificity for this kind of thrombus [4]. The underlying factors leading to RV thrombus formation may be the same as in LV thrombus formation, i.e., mechanical dysfunction related to CM or ischemia, or they may be a part of venous thromboembolism and present as a floating right heart thrombus (FRHTS) in transition to lungs [5,6]. RV thrombi can be classified into type A and type B. Type A are slender, extremely mobile snake- or worm-like thrombi and are a part of venous thromboembolism or FRHTS. Type B could be polymorphic globular or of varying shape and more or less immobile. Some could be intermediate. Type A, or FRHTS, has a poor prognosis and worse mortality rates than type B thrombi [7-9]. LV thrombi can lead to systemic arterial thromboembolism, including but not limited to strokes, MI, ischemic limbs, and intestinal ischemia. RV thrombi could be complicated by pulmonary embolism or paradoxical strokes and could be a reason for mechanical cardiac arrest.

The treatment varies according to location and associated complications. Protuberant or mobile LV thrombus has a higher rate of embolization than mural thrombus. Both are treated with oral anticoagulation (OAC). Warfarin is commonly used, but some studies have shown direct-acting oral anticoagulants (DOAC) to be non-inferior to warfarin [4]. Nevertheless, there are no organizational guidelines that recommend DOAC over warfarin. Duration is at least three months in all cases, including post-MI LV thrombus and CM-related thrombi. A similar or higher-sensitivity imaging, such as MRI, should be obtained to document the persistence or resolution of the thrombus. Continuation of OAC beyond three months should involve shared decision-making factoring in other variables such as persistence of thrombus, persistently low EF, presence of other risk factors, and complications such as malignancy and recurrent strokes, amyloidosis, bleeding, etc. There is no conclusive evidence to support the prophylactic use of OAC to prevent LV thrombus formation except in some specific CMs and LV dysfunction, such as LV non-compaction, peripartum CM, and Takotsubo CM, which is usually decided on a case-by-case basis [4,10-14].

Other infrequent interventions for treating LV thrombi include fibrinolysis and surgical thrombectomy. Both these approaches are not recommended as primary treatment for LV thrombi but may be used if another indication exists, like an acute stroke with an evident LV thrombus or open-heart surgery for multi-vessel coronary artery disease (CAD) or valvular surgery [15-18]. Similarly, RV thrombus is treated according to accompanying complications and underlying risks leading to thrombus formation. If it develops in the setting of cardiac risk factors such as CM and with no concurrent venous thromboembolic disease, it can be treated with anticoagulation, similar to LV thrombi. If it is a thrombus in transit, it may be an emergency, depending on the size and number, due to impending cardiac arrest. Treatment options, in this case, include anticoagulation, thrombolysis (intravenous or catheter-directed thrombolysis), mechanical thrombectomy (e.g., suction thrombectomy), and surgical embolectomy. There are no established guidelines for such cases, but observational studies suggest that thrombolysis or surgical thrombectomy may be more effective than anticoagulation alone in managing these patients [8]. The thrombus' size and the initial severity of pulmonary embolism also influence this decision [19].

**Figure 3:**
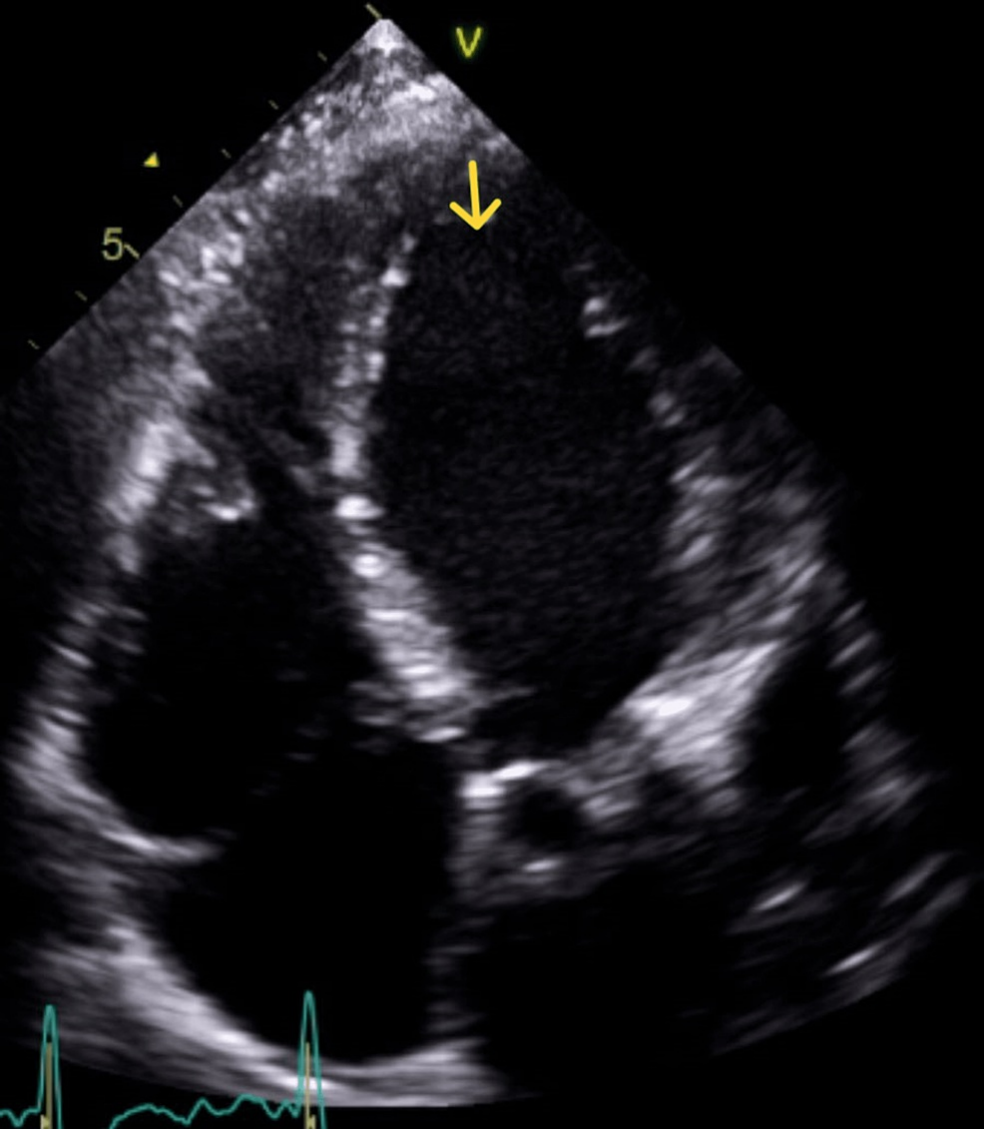
A repeat echocardiogram showing the resolution of thrombus

Our patient had a biventricular thrombus in the setting of severe cardiac dysfunction and a recent myocarditis episode. He was treated with OAC alone with documented complete resolution of biventricular thrombi within three months. However, he was continued on OAC due to persistent low EF. Due to logistics and compliance issues leading to poor INR control, he was switched from warfarin to rivaroxaban (20 mg) seven weeks from hospital discharge. A repeat echocardiogram at 12 weeks from the initial diagnosis of biventricular thrombosis showed complete resolution of thrombi with persistent low EF of less than 20% (Figure 3). Due to persistent low EF, OAC was continued. The patient is currently being evaluated for a heart transplant and has a left ventricular assist device (LVAD).

## Conclusions

Biventricular thrombus formation can occur even in the absence of hypercoagulability, contrary to what was previously believed. Disturbed cardiac mechanics can lead to intracardiac thrombus formation in both ventricles. Regardless of the etiology, it should be treated according to the location and associated complications, such as venous thromboembolism, hemodynamic collapse, obstructive shock, or major stroke. Interventional studies are needed to compare DOACs with warfarin use in such patients.
